# Extra-metabolic energy use and the rise in human hyper-density

**DOI:** 10.1038/srep43869

**Published:** 2017-03-02

**Authors:** Joseph R. Burger, Vanessa P. Weinberger, Pablo A. Marquet

**Affiliations:** 1 Department of Biology, University of North Carolina, Chapel Hill, USA; 2 North Carolina Museum of Natural Sciences, Raleigh, USA; 3 Departamento de Ecología, Facultad de Ciencias Biológicas, Pontificia Universidad Católica de Chile, Alameda 340, Santiago, Chile; 4 Instituto de Ecología y Biodiversidad (IEB), Santiago Chile; 5 The Santa Fe Institute, 1399 Hyde Park Road, Santa Fe, NM, USA; 6 Laboratorio Internacional de Cambio Global (LINCGlobal) & Centro de Cambio Global UC, Pontificia Universidad Católica de Chile, Alameda 340, Santiago, Chile

## Abstract

Humans, like all organisms, are subject to fundamental biophysical laws. Van Valen predicted that, because of zero-sum dynamics, all populations of all species in a given environment flux the same amount of energy on average. Damuth’s ’energetic equivalence rule’ supported Van Valen´s conjecture by showing a tradeoff between few big animals per area with high individual metabolic rates compared to abundant small species with low energy requirements. We use metabolic scaling theory to compare variation in densities and individual energy use in human societies to other land mammals. We show that hunter-gatherers occurred at densities lower than the average for a mammal of our size. Most modern humans, in contrast, concentrate in large cities at densities up to four orders of magnitude greater than hunter-gatherers, yet consume up to two orders of magnitude more energy per capita. Today, cities across the globe flux greater energy than net primary productivity on a per area basis. This is possible by importing enormous amounts of energy and materials required to sustain hyper-dense, modern humans. The metabolic rift with nature created by modern cities fueled largely by fossil energy poses formidable challenges for establishing a sustainable relationship on a rapidly urbanizing, yet finite planet.

All populations, including humans, are sustained by fluxes of energy and materials from a finite environment. Physical constraints on biological design result in ubiquitous and predictable allometric scaling laws^1^. These take power law form where some trait of interest (*R*), scales with body size (*M*),


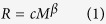


where *β* is the exponent and *c* is the intercept. Metabolic scaling theory predicts quarter-power exponents for rates and quantities across many levels of biological organization^2^,^3^ including whole organism and mass-specific metabolic rates, which scale as *β* ≈ ¾ and *β* ≈ −¼, respectively. Allometric parameters (i.e., intercept and slope) can be predicted theoretically and evaluated empirically to form a quantitative framework to carry out meaningful comparisons across scales from cells and organisms^3^ to human societies^4^,^5^. Using this framework as a reference we aimed to understand unique aspects of human ecology and to quantify the extent to which the human species has departed from the energetic constraints that keep all other species in check.

An important ecological implication of metabolic scaling is the inverse relationship between body size and density. Because individual metabolic rate (*E*_*i*_) scales predictably with size^2^


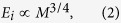


the maximum number of individuals per unit area (*D*_*max*_), scales as the inverse of individual energy demands^6^,^7^


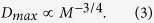


The result is a tradeoff between size and abundance. The scatter around the best-fit line reflects environmental fluctuations linked to biotic interactions and temporal and spatial heterogeneity in resource availability, which appear as positive and negative deviations from maximum densities. Abundance *per se* is not limited by body mass but instead by the energy required to support an individual, *E*_*i*_, of a given body size. Rearranging the allometric relationships in Eqn 2 and 3, theory predicts that


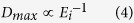


and population energy flux, *E*_*p*_, calculated as the product of *E*_*i*_ · *D*_*max*_, is invariant across species, with *β* = 0


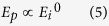


This ‘energetic-equivalence rule’ (EER^6^,^7^) links individual metabolic requirements to population energy use in space and time. This is consistent with the existence of a zero sum game for energy use as predicted by Van Valen^8^,^9^ and quantified in local mammal communities^10^. Unique to industrial humans, however, is the capacity to harness extra-metabolic energy in the form of renewables and fossil fuels to power modern agricultural-technological-industrial lifestyles. Among human societies, individual energy consumption varies from ~120 watts of biological metabolism — the equivalent of ~2500 kilocalories per day — in hunter-gatherers to more than 10,000 watts in the most developed nations^5^,^11^,^12^. So clearly, humans have deviated from other species in their energy use.

## Results

**Figure 1:**
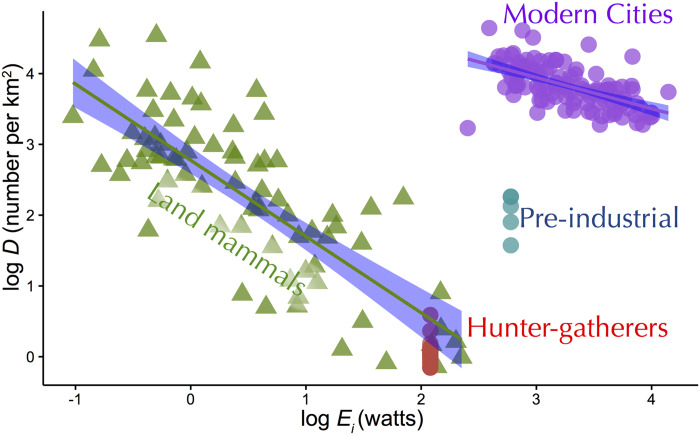
The log of population density (*D*) as a function of log individual energy use (*E*_*i*_) for human populatons (circles) and other land mammals (triangles). Red circles represent vegetarian hunter-gatherers (n = 31), blue circles are pre-industrial societies (n = 4), purple circles are modern cities (n = 163), and green triangles are other herbivorous land mammal species (n = 74). Note that the slope for cities is shallower than herbivorous land mammals, which support theoretical predictions of −1. See supplemental materials for additional details and data sources.

Figure 1 shows that herbivorous land mammals support theoretical predictions where density decreases proportionally with individual energy requirements (slope = −1.08; 95% CI: −0.88, −1.27). Hunter-gatherers, in contrast, occur at densities lower than expected based on other land mammals (ANOVA, F-interaction = 6.37, p < 0.001, Tukey post-hoc test). The trophic position, where an organism feeds in the food web, explains additional variation in the densities of land mammals and hunter-gatherers where densities decrease with higher trophic levels (Supplemental materials; Table S1).

Across cities, density scales negatively with increasing per capita energy requirements consistent with theoretical predictions and similar to the scaling of land mammals. However, modern city dwellers occur at densities that are four orders of magnitude greater than hunter-gatherers and other land mammals (Fig. 1; Table S1) even though they consume one to two-orders of magnitude greater energy per capita. The highest density city in our data (Dhaka, Bangladesh with 44,000 ind/km^2^) now surpasses the highest density wild rodent (Townsend’s vole with 34,349 ind/km^2^). The slope for urban humans (−0.44; 95% CI: −0.32, −0.55) is shallower than the theoretical expectation (slope of −1) and estimated empirical slope from land mammals (Table S1).

**Figure 2:**
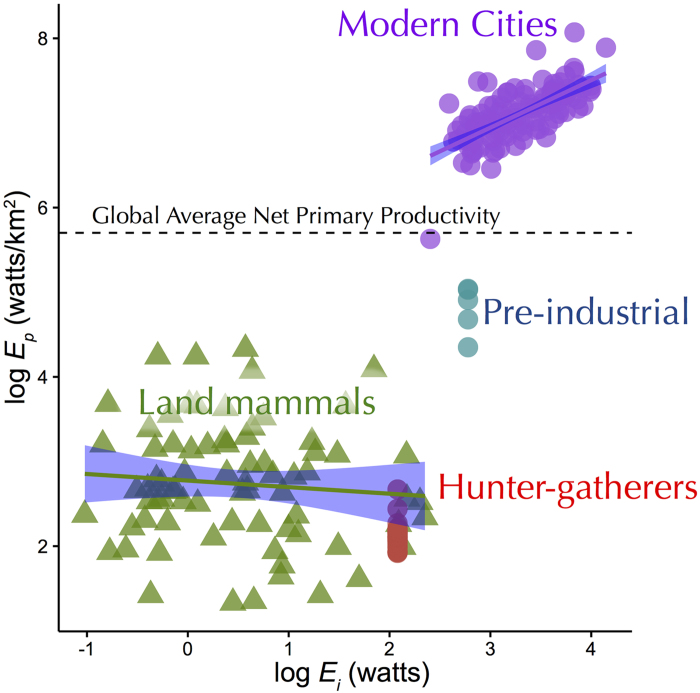
Population energy flux (*E*_*p*_) as a function of individual energy use (*E*_*i*_) for human populations (circles) and other land mammals (triangles). Red circles represent vegetarian hunter-gatherers (n = 31), blue circles are pre-industrial societies (n = 4), purple circles are modern cities (n = 163), and green triangles are other herbivorous land mammal species (n = 74). *E*_*p*_ is estimated as the product of density (individuals/km^2^) and *E*_*i*_ (watts). Note that population energy use for herbivorous land mammals does not vary with individual energy use supporting theoretical expectations (slope = 0), whereas urban cities increase (positive slope). The dashed line represents the terrestrial average net primary productivity for the planet from^23^.

Consistent with Van Valen’s zero-sum prediction and the EER, the energy flux by herbivorous land mammal populations (10^−1^ to 10^4^ watts/km^2^) is invariant with individual energy use (Fig. 2) with a slope indistinguishable from 0 (−0.08, 95% CIs: −0.28, 0.12). Energy flux by hunter-gatherers range from 10^2^ to 10^3^ watts/km^2^ and is lower than other land mammals (ANOVA, F-value interaction = 10.01, p < 0.001, Tukey post-hoc test). Members of pre-industrial societies fluxed greater energy (10^4^ to 10^5^ watts/km^2^) than hunter-gatherers and other land mammals on average but less than cities. Energy flux in modern cities ranges from 10^5^ to 10^8^ watts/km^2^ and surpasses global primary productivity on land (10^5^ watts/km^2^ global avg). Unique to urban humans is the positive relationship between population energy flux and per capita energy requirements (slope = 0.56 [95% CI: 0.44, 0.68]), whereas other land mammals show a slope indistinguishable from 0 as theory predicts (Fig. 2). See supplemental for additional analyses by trophic levels.

## Discussion

Just like all mammals, hunter-gatherers are faced with the challenge of meeting metabolic demands from the local environment to power their lifestyles and sustain their populations. Pre-industrial societies lived at greater densities than hunter-gatherers through the use of agriculture. Humans in modern cities live at densities much greater than those in hunter-gatherers and pre-industrial societies and consume up to two orders of magnitude greater energy per capita than caloric needs alone. This shows that the rapid rise in human densities, which has occurred in less than 10,000 years, is coincident with innovations in food production and extra-metabolic energy use from renewable and fossil fuels^13^.

One salient characteristic of complex human social systems is our ability to copy the behavior of others to propel a cumulative cultural evolution (CCE^14^). It is easy to see that this process likely speeds up with greater population density and information flow as a result of greater energy throughput. The positive slope for population energy flux with per capita energy use in Fig. 2 is a consequence of a shallower slope in Fig. 1. This suggests that increased per capita energy use – which is tightly coupled to economic growth^5^,^12^ – results in economies of scale by packing more individuals in a given area. It is hypothesized that population density—through its effect on CCE—is a major driver of innovation^4^,^15^ and increased social complexity^16^. It is possible that these processes generate positive feedbacks and a runaway process of cultural niche construction^17^ contributing to the rapid divergence of humans from other species and the rise in human densities from hunter-gatherers to agriculturalists to modern cities. We propose that this unique aspect of human ecology is a result of the Malthusian-Darwinian dynamic that drives species to maximize power when innovations allow^18^.

Throughout this process human societies have become increasingly decoupled from local environmental constraints and uncertainties. Technologies that increase resource production and global trade networks that offset imbalances in resource supply and demand provide an enormous buffer from local environmental constraints and perturbations such as drought and other human and natural disasters^19^. However, hyper-dense cities are only possible by the fluxes of vast quantities of energy, materials, and information across city boundaries in order to offset resource sinks and maintain dense, urban lifestyles^20^. The energy flux required to sustain hyper-dense cities now surpasses baseline levels of net primary productivity on a per area basis (Fig. 2) and is largely (~85% globally) in the form of carbon-based fossil fuels^5^. Increasing scarcity of essential resources including fossil fuels^5^, water^21^, and nutrients such as phosphorous^20^ pose formidable challenges for continued urbanization and high-density cities. The cumulative impact of hyper-dense cities may surpass planetary tipping points^22^ having rippling effects at multiple scales.

Our human macroecological approach offers illuminating insights into how humans have rapidly diverged from other species in the course of our unique CCE. Through the use of extra-metabolic energy, modern humans have escaped the energetic constraints of primary productivity that are imposed on all other species. Our approach also highlights the extraordinary densities that humans have obtained through the continued capacity to harness energy reserves from the planet in the form of sunlight^23^ to energy stored on geological time scales^24^. Whether density-dependent innovations will continue to outpace resource constraints on human population is uncertain. What is certain is that the steep metabolic rift with nature^25^,^26^ created by the vast extra-metabolic energy subsidies required to support growing, hyper-dense cities poses formidable challenges to achieving sustainability in a post-fossil fuel world.

## Methods

### Data

We use global data from ecological, archeological, demographic, and economic sources (Supplemental data). Data for metabolic rates and densities for 249 land mammals are from the PANTHERIA species-level database^27^. Densities for 339 hunter-gatherers are available in Binford’s comparative ethnographic and environmental database^28^. Hunter-gatherer lifestyles are powered by biological metabolism estimated to be 120 watts (~2500 kilocalories/day) based on established allometries for primates (following refs 5 and 11). Densities for pre-industrial societies (n = 4) are from^29^ and energy use estimated at 600 watts^11^.

The EER applies to primary consumers^6^,^7^, so we plot herbivorous land mammals (n = 74) and hunter-gatherers that obtain greater than two-thirds of their diet from plants (i.e., gatherers, N = 31) in Figs 1 and 2. It is well known that species at higher trophic levels occur at lower densities (e.g., refs 30 and 31) and hunter-gatherers are no exception with gatherers occurring at higher densities than omnivores, which occur at higher densities than carnivores (See supplemental materials for additional trophic analyses).

It has been suggested that the EER predicts maximum animal densities^32^,^33^. So, we use the densest city per country for which data are available from Demographia World Urban Areas (demographia.com; website has updated lists). The data consists of censuses conducted between 2000 and 2014. In contrast to hunter-gatherers, urban human lifestyles are powered by both biological metabolism and extra-metabolic energy in the form of renewables and fossil fuels^11^,^34^. However, a global database on metabolic (i.e., caloric) and extra-metabolic energy use for cities is not available. So we estimated per capita energy use by combining country-level data on food consumption per country (kcal per capita per day) from the Food and Agriculture Organization from of the United Nations (FAO) (http://faostat3.fao.org/) with extra-metabolic energy from The World Bank Indicators (http://data.worldbank.org/indicator; website has updated data). We use only one city (the densest) per country in our analyses although more cities are listed in the demographia dataset. This assumes greater variation among countries than within, which is supported by studies showing that resource use and waste production scale linearly (e.g., constant per capita) with city size within countries^4^,^35^.

### Statistical Analyses

We determined the relationship between population density and per capita energy requirements separately for land mammals and urban humans using ordinary least squares regression of log transformed variables. We compared 95% confidence intervals of allometric parameters from linear models of the log-log relationships for land mammals and modern cities in order to evaluate theoretical expectations (slope = −1).

We conducted Analysis of Covariance (ANCOVA), with per capita energy use as the covariate, density as the dependent variable, and trophic level or human versus land mammal as fixed factors. Comparisons were made between: (*i*) land-mammals by trophic levels (ANCOVA), (*ii*) land-mammals and hunter-gatherers considering trophic level as a second factor (ANOVA), and (*iii*) land mammals (with trophic level as a cofactor) versus urban humans (ANCOVA).

We calculated population energy requirements (*E*_*p*_) as the product of population density and per capita energy use to test the EER that all populations flux the same amount of energy per unit area as a consequence of the zero-sum. We conducted ANCOVAs and ANOVAS similar to above with *E*_*p*_ as the dependent variable.

## Additional Information

**How to cite this article:** Burger, J. R. *et al*. Extra-metabolic energy use and the rise in human hyper-density. *Sci. Rep.* **7**, 43869; doi: 10.1038/srep43869 (2017).

**Publisher's note:** Springer Nature remains neutral with regard to jurisdictional claims in published maps and institutional affiliations.

## Supplementary Material

Supplementary Materials

Supplementary Dataset 1
