# Supplementary material for: Extra-metabolic energy use and the rise in human hyper-density
Source: Sci Rep. 2017 Mar 2;7:43869. doi: 10.1038/srep43869 (PMC5333137; doi:10.1038/srep43869)
Supplement: Supplementary Materials [file srep43869-s1.pdf]

Supplementary Materials for

Extra-metabolic energy use and the rise in human hyper-density

Joseph R Burger*[1,2], Vanessa P Weinberger[3,4], Pablo A Marquet[3,4,5,6]
*Correspondent email: jrburger@email.unc.edu

[1] Department of Biology, University of North Carolina, Chapel Hill, USA

[2] North Carolina Museum of Natural Sciences, Raleigh, USA

[3] Departamento de Ecología, Facultad de Ciencias Biológicas, Pontificia Universidad Católica de Chile, Alameda 340, Santiago, Chile

[4] Instituto de Ecología y Biodiversidad (IEB), Santiago Chile

[5] The Santa Fe Institute, 1399 Hyde Park Road, Santa Fe, NM 87501, USA

[6] Laboratorio Internacional de Cambio Global (LINCGlobal) & Centro de Cambio Global UC, Pontificia Universidad Católica de Chile, Alameda 340, Santiago, Chile

Supplemental Materials include:

Trophic analyses
Additional results
Figures S1 and S2
Tables S1 and S2
References
Data table

Trophic level analyses

Trophic energetics are known to influence abundance in addition to body size (e.g.,[1–4]). So we conducted a separate analysis comparing density and energy use in land mammals and hunter-gatherers by trophic levels (Fig S1 and S2). Discrete trophic levels for land mammals are from the PANTHERIA species-level database[5]. Percent diet from hunting, fishing, or gathering are available for hunter-gatherers in Binford[6]. We classified meat-eaters with ≥ 66% of diet from hunting and fishing as carnivores (n=163), mixed-diets with ≥ 33% but < 66% from plants as omnivores (n=145), and ≥ 66% of diet from plants as herbivores (n=31).

We determined the relationship between population density and per capita energy requirements separately for land mammals and urban humans using ordinary least squares regression of log transformed variables similar to [7–9]. We compared 95% confidence intervals of the slopes from linear models of the log-log relationships for land mammals and modern cities in order to evaluate theoretical expectations (slope = -1) from empirical observations.

To test for differences in density among trophic levels for land mammals, we conducted Analysis of Covariance (ANCOVA), with per capita energy use as the covariate and trophic level as a fixed factor. To compare land mammals and hunter-gatherers, we also conducted Analysis of Variances (ANOVA) with trophic level as a second factor. To compare modern cities with other land mammals we conducted a second ANCOVA similar to the above analysis using only land mammals.

To test the null hypothesis that all populations flux the same amount of energy per unit area as a consequence of the zero-sum, we calculated population energy requirements as the product of population density and per capita energy use. We conducted ANOVAs and ANCOVAs similar to above analyses but with population energy flux (E_p) as the response variable.

Additional results

Density and energy use by trophic levels

Density decreases with higher trophic levels in land mammals and hunter-gatherers are no exception. The slopes for each trophic level for land mammals (Fig S1) are significantly different (Table S1, ANOVA, F-value interaction= 6.49, p-value < 0.001, Tukey post-hoc test). The slope for herbivores is statistically indistinguishable from theoretical expectations of -1 (-1.08 [CI: -0.879, -1.27]) (Table S1) and higher trophic levels show significantly steeper slopes than theory predicts: -1.29 for omnivores and -1.63 for carnivores (Table S1). This is likely due to the greater uncertainty in food availability in space and time for organisms at higher trophic levels and has been discussed elsewhere[3,4]. Shifts in the elevation (intercepts) of slopes for herbivores, omnivores, and carnivores is roughly an order of magnitude and corresponds to an approximate 10% energy transfer between trophic levels[1].

A two way ANOVA comparing land mammals and hunter-gatherers by trophic level shows a significant interaction between factors (ANOVA, F-value interaction= 6.37, p-value < 0.001), with each hunter-gatherer, by trophic level acquiring densities significantly lower than their land mammal counterparts (Tukey post-hoc test, p-values < 0.001 for all level comparisons). Hunter-gatherers also show significant trophic effects (ANOVA, p-value < 0.001 for all group comparisons, Tukey post-hoc test), with predominately vegetarians achieving the highest densities, followed by omnivores and carnivores (Figure S1). It is important to note that none of the 339 hunter-gatherers reported in Binford are exclusively vegetarian which may explain why they occur at lower densities than their land mammal counterparts in all tests. Modern cities are also different from land-mammals when compared to trophic categories (ANCOVA, F-value interaction= 16.06, p-values < 0.001, Tukey post-hoc test) with cities having shallower slopes (Table S1).

Energy flux and trophic levels

Comparing energy flux per unit area among land mammals by trophic level we find that herbivorous land mammals have a slope indistinguishable from 0 (-0.08 [CI: -0.28,0.12]), as expected by theory, and flux greater energy than predominately vegetarian hunter-gatherer (ANOVA, F-value interaction=6.37, p-values<0.001, Tukey post-hoc test; Table S2). For land mammals, higher trophic levels show significantly steeper slopes (ANCOVA, F-value interaction= 6.49, p-value < 0.001, Tukey post hoc test) and population energy flux in omnivorous and carnivorous hunter-gatherers are indistinguishable from their land mammal counterparts (ANOVA, F-value interaction= 10.01, p-values<0.001, Tukey post-hoc test). When comparing land mammals to modern cities, we find that modern cities are significantly different from all trophic levels (ANCOVA, F-value interaction= 16.05, p-value<0.001, Tukey post-hoc test). This is evident by the unique positive slope compared to other land mammals in Figure 2.

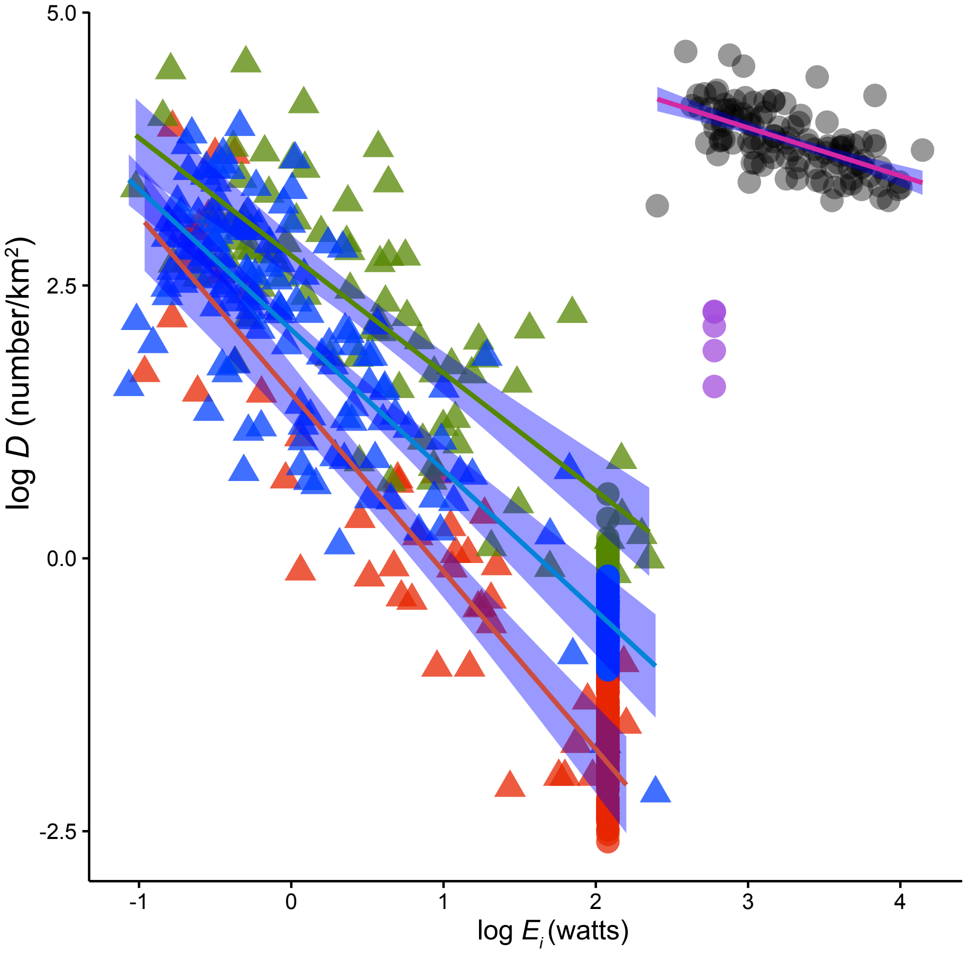

Figure S1. Log density versus log individual energy use for humans (circles) and other land mammals (triangles) distinguished by trophic level: red = carnivores, blue = omnivore, green = herbivores, purple = pre-industrial agriculturalists, grey = city dweller.

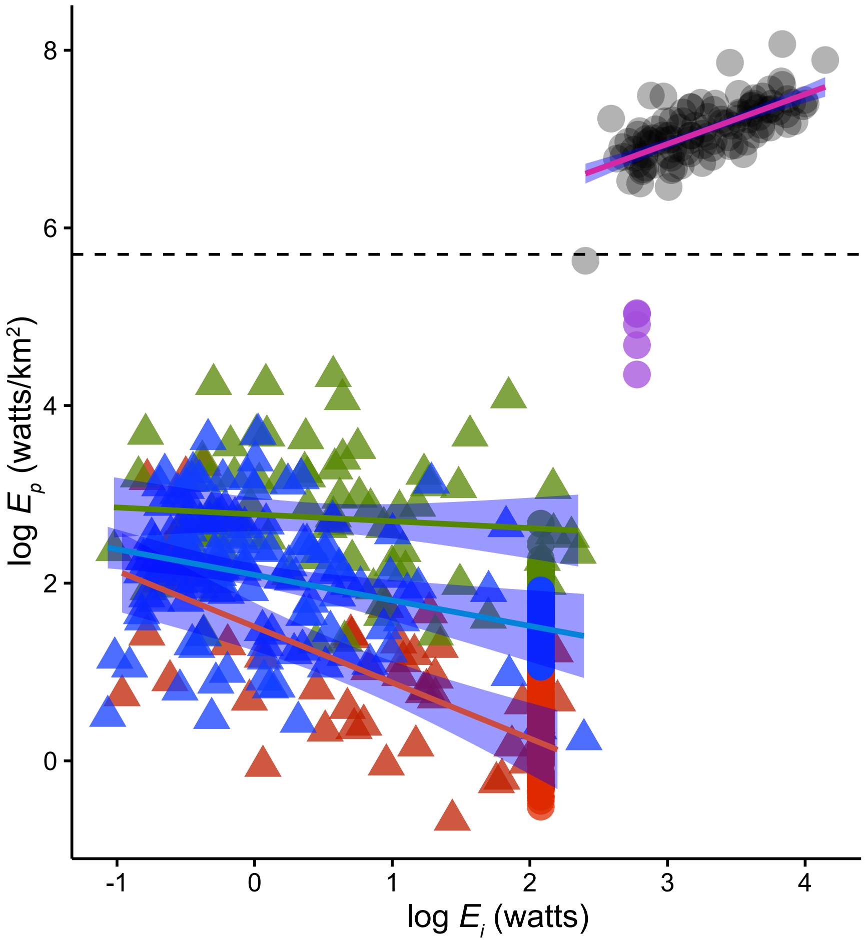

Figure S2. Population energy flux (the product of individual energy use and density) versus individual energy use for human societies (circles) and other and land mammals (triangles) distinguished by trophic level: red = carnivores, blue = omnivores, green = herbivores, purple = pre-industrial agriculturalists, grey = city dweller. The dashed line represents the terrestrial average net primary productivity for the planet.

Table S1: Scaling parameters with 95% confidence intervals by category for Fig S1. Herbivore slope is indistinguishable from theoretical expectations of -1.

Category	R- adjusted	P-value	Slope	Intercept	Degrees of freedom
**Cities**	0.33	2.04e-11	-0.44 [-0.32, -0.56]	5.26 [4.88, 5.42]	109
**Herbivores**	0.62	2.2e-16	-1.08 [-0.879, -1.27]	2.77 [2.58, 2.96]	73
**Omnivores**	0.59	2.2e-16	-1.29 [-1.1, -1.48]	2.09 [1.97, 2.21]	127
**Carnivores**	0.80	2.2e-16	-1.63 [-1.39, -1.87]	1.51 [1.25, 1.76]	43
**All land mammals**	0.57	2.2e-16	-1.30 [-1.16, -1.44]	2.19 [2.07, 2.31]	247

Table S2: Slopes and intercepts with (95% confidence intervals) for Plot 2. Zero-sum theory predicts a slope of zero and is supported by herbivores.

Category	R-adjusted	P-value	slope	intercept	Degrees of freedom
**Cities**	0.45	5.47e-16	0.56 [0.44, 0.68]	5.27 [4.89, 5.68]	109
**Herbivores**	-0.01	0.439	-0.08 [-0.28, 0.12]	2.78 [2.60, 2.84]	73
**Omnivores**	0.01	0.0031	-0.29 [-0.49, -0.02]	2.09 [1.97, 2.21]	127
**Carnivores**	0.37	5.05e-06	-0.63 [-0.87, -0.39]	1.51 [1.25, 1.76]	43
**All land mammals**	0.06	2.95e-05	-0.30 [-0.44, -0.16]	2.19 [2.07, 2.31]	247

References

1. Lindeman, R. L. The trophic-dynamic aspect of ecology. *Ecology.* **23,** 399–417 (1942).
2. Pablo A, M., Sergio A, N. & Juan C, C. Scaling Population Density to Body Size in Rocky Intertidal Communities. *Science.* **250,** 1125–1127 (1990).
3. Carbone, C. & Gittleman, J. L. A common rule for the scaling of carnivore density. *Science.* **295,** 2273–2276 (2002).
4. Marquet, P. A. Of predators, prey, and power laws. *Science.* **295,** 2229–2230 (2002).
5. Jones, K. E. *et al.* PanTHERIA: a species-level database of life history, ecology, and geography of extant and recently extinct mammals. *Ecology.* **90,** 2648–2648 (2009).
6. Binford, L. R. *Constructing frames of reference: an analytical method for archaeological theory building using ethnographic and environmental data sets*. (Univ of California Press, 2001).
7. Moses, M. E. & Brown, J. H. Allometry of human fertility and energy use. *Ecology Letters.* **6,** 295–300 (2003).
8. Burger, O., DeLong, J. P. & Hamilton, M. J. Industrial energy use and the human life history. *Scientific Reports.* **1,** (2011).
9. DeLong, J. P., Burger, O. & Hamilton, M. J. Current demographics suggest future energy supplies will be inadequate to slow human population growth. *PLoS One.* **5,** e13206 (2010).
